# Genomics in Plant Viral Research

**DOI:** 10.3390/v14040668

**Published:** 2022-03-24

**Authors:** Solomon Maina, Brendan Rodoni

**Affiliations:** 1 New South Wales Department of Primary Industries, Biosecurity & Food Safety, Elizabeth Macarthur Agricultural Institute, Woodbridge Road, Menangle, NSW 2568, Australia; 2 Microbial Pests & Diseases, Agriculture Victoria Research, AgriBio, Bundoora, VIC 3083, Australia; brendan.rodoni@agriculture.vic.gov.au; 3 School of Applied Systems Biology, La Trobe University, Bundoora, VIC 3083, Australia

Plant viruses constitute a large group of pathogens causing damaging diseases in many agricultural and horticultural crops around the world. As such, management strategies including advanced diagnostics are an integral part of minimizing crop viral diseases. Integrating innovative rapid diagnostic tools offers an effective pathway for disease management for sustainable food production. They allow the detection of new and re-emerging threats, especially as new cropping systems evolve, particularly in response to climate change. In addition, in several instances, advanced genomic diagnostics tools such as high-throughput sequencing (HTS) offer biological insights into the genomic structure of a complex viral pathogen, which is critical to new pathogen diagnostic design and ongoing development and implementation of viral disease management strategies. Eight original articles in this Special Issue provide comprehensive information on the immense potential of HTS in unraveling damaging crop viral pathogens and further aspects of current and future principles of crop viral disease management.

Olive (*Olea europaea* L.) is one of the most cultivated fruit crops around the world. It is vegetatively propagated and known to be non-symptomatic when infected by different viruses. Patrick Materatski and colleagues [1] identified *Olea europaea Geminiviridae* (OEGV), isolated in Portuguese olive trees, using HTS. This study described the new OEGV unique features to be distinct from other geminiviruses. Its bipartite genome (DNA-A and DNA-B) is related to Old World begomoviruses (OW) in length, but lacked a pre-coat protein (AV2), a typical feature of New World begomoviruses (NW) [1]. These results suggested that OEGV evolution may have occurred from an ancient OW monopartite begomovirus that lost V2 and C4, and later gained functions on cell to cell movement through the acquisition of a DNA-B component [1]. The findings also found that the invasion of new host cells may have been facilitated by a new polyphagous vector that mediated its introduction to olives. The study also highlighted for the first time the similarities and differences between OW and NW begomoviruses as well as mastreviruses and curtoviruses, indicating potential new evolutionary pathways of the *Geminiviridae* family.

Ornamental plants are sources of viruses and viroids that not only infect ornamental plants, but also serve as reservoirs or alternative hosts for major viruses that infect cultivated crops. In the current globalization, ornamental plants could introduce new viruses into different geographical zones inadvertently [2]. Hang Yin and colleagues [3] revealed the applicability of HTS as an effective tool for screening potential pathogens within ornamental plants that can lead to severe epidemiological risks. Marigold (*Tagetes erecta* L.) is native to Mexico and is cultivated as a commercial garden ornamental, source of dye plants, marigold meal, or for its medicinal value, [4,5,6,7]. The HTS capability revealed mixed marigold virus infections [3], such as broad bean wilt virus-2, turnip mosaic virus, and two novel viruses tentatively named marigold mosaic virus (potyvirus) and marigold cryptic virus (deltapartitivirus).

Chinese jujube (*Ziziphus jujuba* Mill.) is a native fruit crop in China. Jiashu Guo et al. [8], described the sequences of three jujube yellow mottle-associated virus (JYMaV) isolates, with one being a variant. These viruses were determined by HTS using small RNA and rRNA-depleted RNA approaches coupled with RT-PCR [8]. Comparison of these sequences together with sequences of the viral RNA segments derived by RT-PCR revealed that genetic diversity was present in the virus populations. The research revealed a high sequence variation occurrence at the non-translational regions of each of the viral genomic segments [8]. In addition, their findings found that P5 encoded in the viral RNA5, displayed a nuclear localization feature differing from the plasmodesma subcellular localization of the virus movement protein (P4). The bimolecular fluorescence complementation (BiFC) assays proved an interaction between P4 and P5 proteins [8]. This study constitutes the first report of area-wide occurrence and the molecular diversity of JYMaV in jujube trees grown at Aksu in Xinjiang.

Liqin Tu and colleagues [9], studied tomato mottle mosaic virus (ToMMV), which belongs to the *Virgaviridae* family and causes devastating losses in tomatos. The research involved the isolation and cloning of a full-length genome of a Chinese isolate (ToMMV-LN) from a naturally infected tomato (*Solanum lycopersicum* L.) [9]. An infectious cDNA clone of ToMMV was constructed by a homologous recombination approach. After agroinfiltration, *Nicotiana benthamiana*, tomato and pepper developed severe symptoms revealing strong infectivity [9]. The study demonstrated that the cDNA infectious clones were successfully constructed and proved to be of high pathogenicity. This practical approach of constructing infectious cDNA clones proved to be a powerful tool for investigating the characteristics of ToMMV. The present results are significant in understanding pathogenic molecular interactions between host and existing infectious viruses, and the mechanisms underlying viral infection.

Rice (*Oryza sativa*) is a major food crop to almost a half of the global population. The study of Chao et al. [10] used a backcrossing strategy to integrate a dominant brown planthopper (BPH) resistance gene Bph3 into a high-yield and BPH-susceptible indica rice variety. BPH is a major threat to rice production, but they found their approach enhances BPH resistance significantly. According to the study [10], when Bph3-carrying backcross healthy lines were infested with BPH, they depicted sterile characteristics such as panicle enclosure and failure of seed production at maturity stage, probably due to the BPH viral infections [10]. To ascertain the observed sterile symptoms that might be BPH mediated viral infections, HTS was used to characterize viruses present [10]. HTS revealed eight novel virus species and five known viruses, including a highly divergent virus that clustered within an unclassified family. The study also investigated plant antiviral responses using small RNA sequencing, which revealed an abundance of virus-derived small interfering RNAs in sterile rice plants, due to immune responses in rice plants [10]. 

Jones and colleagues [11], reviewed virus diseases of cereals and oilseeds crops in Australia. The comprehensive study emphasized future threats, managing virus diseases, increasing the spectrum of insecticide resistance, resistance-breaking virus strains, changes in epidemiology, virus and vector impacts arising from climate instability and insufficient industry awareness of virus diseases. A major highlight discussed challenges associated with crop virus management such as resistance breaking. For instance, turnip mosaic virus in canola is controlled effectively by planting crop cultivars with virus resistance genes, but there exists a challenge of resistance-breaking virus strains that can overcome the present crop resistance genes [11,12]. The review recommended monitoring of both cereal and oilseed crops and nearby weeds to search for resistance-breaking virus strains [11] and avoiding their introduction by different routes from nearby countries or other parts of the world. 

Jones and colleagues [11] also reinvigorated the vital role of biosecurity measures in pre- and post-border activities, considering the magnitude of the threat posed to Australian cereal and oilseed crops by exotic viruses if they become established in the country. The authors acknowledge that, globally, Australian plant biosecurity measures are among the strictest, and largely this contributes to the country being free from many damaging virus diseases that exist elsewhere [11]. To sustain this, the authors suggested unceasing research efforts to cope with an uneven changing biological complex of viruses to strengthen the current procedures. Such efforts include continuously incorporating the latest technology capabilities, such as developing agile robust and cost-effective genomic diagnostics tools, and surveillance strategies to avoid establishment of viruses and their vectors within Australia [13].

Wamonje [14] described the COVID-19 pandemic as re-igniting the importance of genomics applications within plant virology, including serving in diagnostics and understanding plant virus transmission to manage viral pandemics. The opinion article reiterated previous viral disease outbreak lessons that apply in plant viruses within developing countries [14]. For example, maize lethal necrosis disease, which severely impacted the yields of maize crops in eastern and southern parts of Africa, was later identified and characterized by HTS, opening an opportunity for its management [15]. The author describes the gap underlying viral diagnostics and the importance of building local capacity for rapid diagnosis of plant viruses in the region [14]. This article also highlighted the shortcomings of agricultural research in Africa, especially the lack of established, sustainable HTS capabilities, which are pivotal in using genetic information to develop cost-effective deployable diagnostics in Africa. Levelling up such capabilities will contribute toward better viral disease management for sustainable food production [14], especially in the sub-Saharan region of Africa. Further, the article highlights the recent viral disease outbreaks posing significant challenges to smallholder farmers within the continent [14], where maize and other tuber crops are commonly referred as staple food, and a source of direct and indirect income for many families. The article applauds the current research progress but calls for establishing a new targeted Research Centre in Africa to support cutting-edge research and capacity building in disease management to secure the future of Africa farming systems from damaging diseases.

A considerable amount of research has been published on the application of HTS in plant virology research. Nonetheless, the greatest challenge of HTS adoption as a routine diagnostic tool is the prohibitive cost associated with its operating [13]. This bottleneck has impeded its implementation in germplasm certification programs. The efforts of Maina et al. [13], developed the first detection approach of direct HTS of targeted multiple plant virus amplicon known as targeted genome sequencing (TG-Seq). The approach simultaneously detected multiple (four) distinct important RNA viral species infecting pulses; pea early browning virus (exotic in Australia), cucumber mosaic virus, bean yellow mosaic virus and pea seed-borne mosaic virus. TG-Seq detected all four virus amplicons within a single-tube reaction. The study sheds light on the tremendous opportunity of TG-Seq as a future highly sensitive and cost effective, targeted HTS approach for detecting multiple plant viruses [13]. Their findings also revealed the deconvolution power of multiple plant viruses via increasing targeted primer panels to enhance broad identification of pathogenic plant viruses across multiple plant samples. This approach is cost-effective compared to entire genome sequencing. These findings have major implications for plant health certification programs and biosecurity management in relation to pathogen entry into Australia and elsewhere.

## Conclusions

HTS technologies have revolutionized plant virology research and allowed unprecedented possibilities in virus and viroid discovery in many agricultural crops. Considering that the prevention and management of plant diseases principally depend on an initial accurate identification of pathogens, continuous development, and optimization of HTS technology to advance rapid and specific detection assays are required. This remains crucial, especially in the scenario where a new biological complex crop pathogen has risen or occurred.
